# Application of intensity-modulated radiotherapy in unresectable poorly differentiated thyroid carcinoma

**DOI:** 10.18632/oncotarget.12785

**Published:** 2016-10-20

**Authors:** Fen Xue, Duanshu Li, Chaosu Hu, Zhuoying Wang, Xiayun He, Yi Wu

**Affiliations:** ^1^ Department of Radiation Oncology, Fudan University Shanghai Cancer Center, Shanghai, China; ^2^ Department of Head and Neck Surgery, Fudan University Shanghai Cancer Center, Shanghai, China

**Keywords:** unresectable poorly differentiated thyroid carcinoma, intensity modulated radiotherapy, chemotherapy, locoregional control, distant metastases

## Abstract

Poorly differentiated thyroid carcinoma (PDTC) is a rare and aggressive malignancy with high rates of invasion and distant metastasis. This study was to explore the ability of intensity-modulated radiotherapy (IMRT) combined with chemotherapy to manage unresectable PDTC. Between February 2011 and April 2012, 5 patients with unresectable PDTC were treated by IMRT at our institution and were included in this analysis. The median radiotherapy dose to the gross tumor volume (GTV) was 66 Gy/33 fractions/6.4 weeks. All patients received chemotherapy, and one patient with tumor compression symptoms had a tracheotomy before treatment. The mean survival time of the 5 patients was 41.6 months. The direct causes of death were distant metastases (40%) and progression of the locoregional disease (20%). In conclusion, IMRT combined with chemotherapy for unresectable PDTC might be beneficial to improve locoregional control. Further new therapies are needed to control metastases.

## INTRODUCTION

Poorly differentiated thyroid carcinoma (PDTC) is a rare malignant tumor that accounts for 1-15% of all thyroid cancers. Despite the relative infrequency of this disease, it is the main cause of death from non-anaplastic follicular cell-derived thyroid cancers [1–6]. The diagnostic criteria of PDTC were controversial until its recognition as a separate entity in terms of architecture and high-grade features by the 2004 World Health Organization classification [7] and the 2006 Turin proposal [8]. Morphologically and clinically, PDTC falls between well-differentiated and undifferentiated (anaplastic) carcinomas, and it carries a better prognosis than anaplastic thyroid carcinomas (ATC) but a worse prognosis than well-differentiated thyroid carcinomas (WDTC). Most patients with PDTC die of metastases and an uncontrolled neck mass [4, 9–11].

Given the recent recognition of PDTC as a separate subtype of thyroid cancer, the staging system for PDTC has not yet been established by the World Health Organization [7]. However, considering its similar biology, PDTC was classified according to the seventh AJCC Cancer Staging Manual using the staging system for WDTC [12].

Due to the lack of morbidity and unifying diagnostic criteria for PDTC, a consensus on the management of PDTC has not been reached. Surgical resection remains the standard of care, and ^131^I therapy is indicated postoperatively if the tumor is radio-avid [3, 4, 6, 13–15]. However, PDTC is an aggressive malignancy with a tendency to invade extensively into adjacent organs, such as the trachea, esophagus, larynx, recurrent laryngeal nerve, prevertebral fascia, blood vessels, soft tissues and muscle. Some patients may even develop regional nodal and distant metastases before diagnosis, which will make surgery very difficult or even impossible. Sanders et al. [3] recommended that external beam radiotherapy (EBRT) combined with chemotherapy may be applicable in unresectable PDTC, but no significant impact on survival has been reported in the limited literature available. Therefore, an innovative therapy is desperately needed. Intensity-modulated radiotherapy (IMRT) is an advanced form of three-dimensional conformal radiotherapy (3DCRT) with high doses given to tumor tissue and low doses given to normal tissues, so that a good therapeutic ratio can be achieved. This article explores the outcomes for the first 5 unresectable PDTC patients treated with IMRT combined with chemotherapy at our institution.

## RESULTS

### Radiotherapy

The treatment regimen of the 5 patients is summarized in Table 1. IMRT was administered to all 5 patients to a median total dose of 66 Gy (range: 64-66). The median overall radiation therapy treatment time was 45 days (range: 41-58), and the median number of fractions was 33 (range: 32-33). Four patients (80%) completed radiotherapy at the prescribed dose of 66 Gy. Patient 1 discontinued radiotherapy early due to experiencing esophagitis (grade II) at dose of 64 Gy. By the end of treatment, CT scans from the 5 patients demonstrated stable disease.

**Table 1:** Treatment regimen

Patient no.	Neoadjuvant chemotherapy	IMRT	Concurrent chemotherapy	Adjuvant chemotherapy
1	GP*1	64Gy/32F/42D	DDP*3	GP*1
2	-	66Gy/33F/41D	TP*2	TP*2
3	TP*2	66Gy/33F/45D	TP*1	TP*2
4	TP*4	66Gy/33F/51D	-	-
5	-	66Gy/33F/58D	TP*2	TP*2

GP: Gemcitabine 1.0g/m^2^ d1,8; Cisplatin 40mg/m^2^ d1-3; 3 weeks as 1 cycle.

DDP: Cisplatin 30mg/m^2^ d1; 1 week as 1 cycle.

TP (Neoadjuvant + Adjuvant): Paclitaxel 135 mg/m^2^ d1; Cisplatin 40 mg/m^2^ on d1-3; 3 weeks as 1 cycle.

TP (Concurrent): Paclitaxel 135 mg/m^2^ d1; Cisplatin 40 mg/m^2^ on d1-3; 4 weeks as 1 cycle.

### Chemotherapy

All patients received chemotherapy, and four patients received chemotherapy concurrently with radiotherapy. Patient 1 received concurrent chemotherapy with cisplatin 30 mg/m^2^/week for 3 cycles, while patient 2, 3 and 5 received concurrent chemotherapy with paclitaxel 135 mg/m^2^ on day 1 and cisplatin 40 mg/m^2^ on days 1-3, repeated on day 28. All five patients were given neoadjuvant or adjuvant chemotherapy every 3 weeks as 1 cycle (Table 1). Of these patients, patient 1 with diabetes was given gemcitabine and cisplatin, and other four patients without diabetes were given paclitaxel and cisplatin. The doses for the drugs were as follows: cisplatin 40 mg/m^2^ on days 1-3, gemcitabine 1000 mg/m^2^ on days 1 and 8 and paclitaxel 135 mg/m^2^ on day 1, every 3 weeks. Adjuvant chemotherapy with the same regimen was administered 28 days after the end of radiotherapy.

### Surgery

Surgical intervention included biopsy in all patients and tracheotomy in patient 1 with tumor compression symptoms. Patient 3 had unresectable local recurrence after the unilateral thyroidectomy within 2 months.

### Toxicities

There were no treatment-related deaths. The most common radiation-related acute toxicity was mucositis of pharynx. RTOG grade I occurred in patient 4, and other four patients had grade II. These four patients needed anti-inflammatory, fluid replacement, and symptomatic treatment for mucosal reaction. The median duration was 6 days (range: 2-9), but none of the patients required tube feeding support. Radiation dermatitis was reported as grade III in patient 4. Patient 1 discontinued radiotherapy early due to esophagitis (grade II) and recovered one month later following treatment of the symptoms. The primary side effects attributed to chemotherapy were hematological and digestive toxicities. Grade III neutropenia and thrombocytopenia were observed in patient 1, and grade IV neutropenia was observed in patient 3. Their symptoms relieved after corresponding treatment. None of the patients had grade III or more nausea and vomiting. There were no severe late adverse effects of the therapy, such as esophageal stenosis, pneumonitis or fibrosis (≥ grade II) in the neck.

### Local control, distant metastasis, and survival

**Figure 1:**
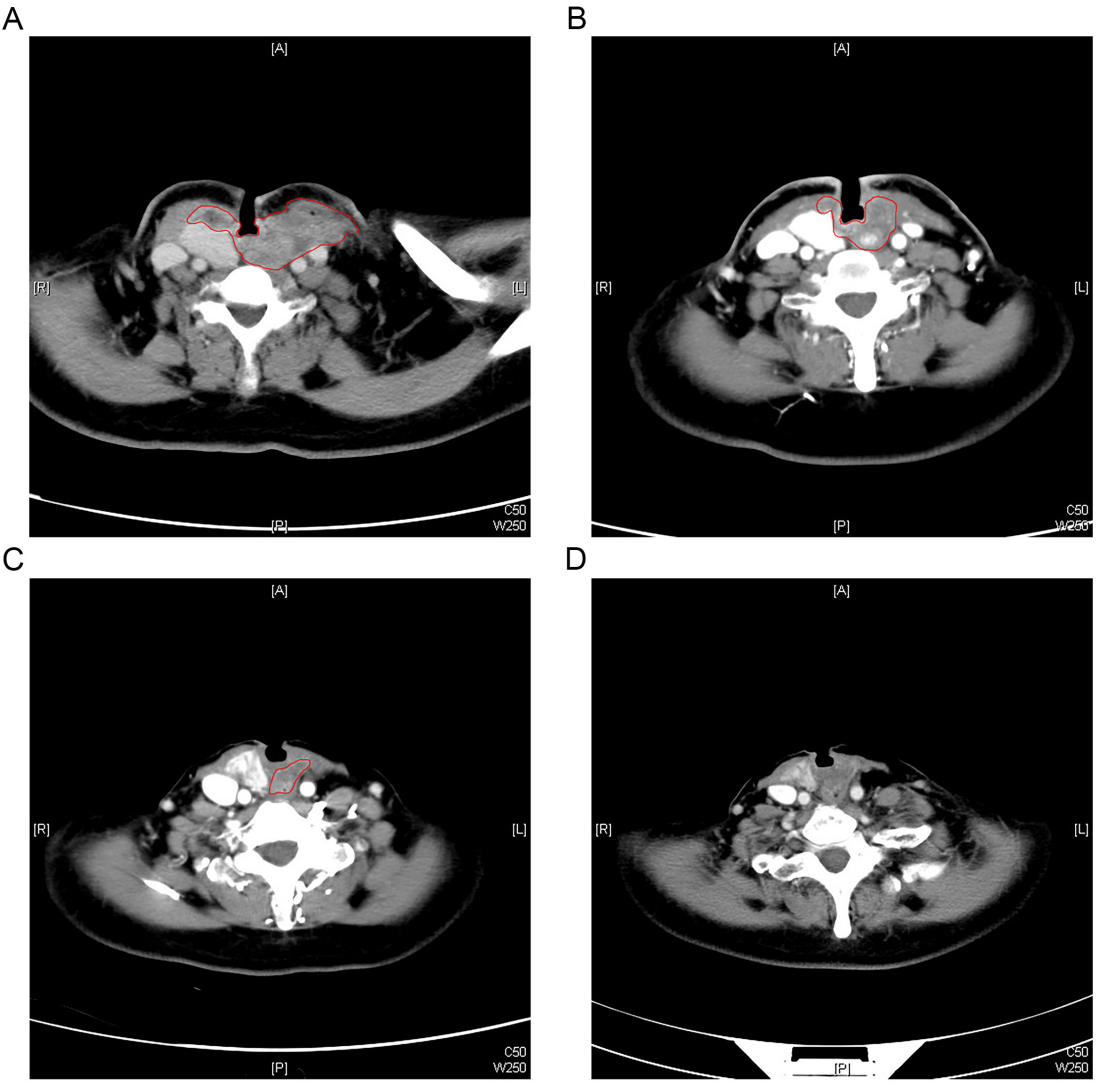
Typical contrast-enhanced CT scan pictures illustrating the evolution of locoregional disease for an example patient **A**. Patient 1 received a tracheotomy before treatment for tumor compression symptoms. **B**. After completion of neoadjuvant chemotherapy and concurrent chemo-radiotherapy, the locoregional disease was in stable disease. **C**. Twenty-one months after treatment, the locoregional disease was in partial remission. **D**. Thirty-four months after treatment, the locoregional disease was in complete remission.

The median follow-up duration was 54 months (range: 8-59), and the mean survival was 41.6 months. The tumor size remained stable in all patients after completing the radiotherapy. At a follow-up of 18 months, the locoregional disease was in partial remission in patients 1-4. However, the patient 3 had progressive disease 3 months later. At a follow-up of 48 months, the locoregional disease was in complete remission in patient 1 and 2 and in partial remission in patient 4. At the latest follow-up, patient 1 and 2 were alive with locoregional disease in complete remission and have been followed for 54 and 57 months, respectively (Figure 1 shows the evolution of the locoregional disease after combined treatment in patient 1). The rest three patients experienced treatment failure and died after 8, 30, and 59 months with locoregional stable disease, progressive disease and partial remission, respectively. The direct cause of death was locoregional failure in one patient and distant metastases in two patients.

The one who failed locally was patient 3. She underwent unilateral thyroidectomy and was treated because of unresectable recurrence. It was noted that she had gross recurrent disease as well as multiple pathologic lymph nodes, both with extracapsular extension. The patient was given a regimen of paclitaxel and cisplatin for 2 cycles before radiotherapy. She then received radiation at a dose of 66 Gy in 33 fractions combined with the same chemotherapy regimen for 1 cycle. Adjuvant chemotherapy was administered for 2 cycles 28 days after completion of the radiotherapy, with the same regimen as well. The locoregional disease was in partial remission 18 months after treatment, but disease progression occurred at a follow up of 21 months. The patient died 30 months after chemo-radiotherapy.

Patient 4 and 5 presented with metastatic lung disease and eventually died from that disease. Like patient 3, they had unresectable disease with gross extrathyroidal extension into adjacent organs and multiple pathologic lymph nodes. Both of the patients received radiation at a dose of 66 Gy in 33 fractions. Patient 4 was given neoadjuvant chemotherapy with paclitaxel and cisplatin for 4 cycles. The patient's locoregional disease was in partial remission after 18 months of treatment, but the patient died at a follow-up of 59 months (locoregional partial remission). Patient 5 was given concurrent and adjuvant chemotherapy with the same regimen for 2 cycles. The patient died at a follow-up of 8 months (locoregional stable disease) (Table 2).

**Table 2:** Treatment outcome

Patient no.	Effect of neoadjuvant chemotherapy	Effect of concurrent chemo-radiotherapy	18 months after treatment	48 months after treatment	Relapse (month)	Follow up (month)	State	Cause of death
1	SD	SD	PR	CR	-	57	Alive	-
2	-	SD	PR	CR	-	54	Alive	-
3	SD	SD	PR	-	21	30	Dead	LR
4	SD	SD	PR	PR	-	59	Dead	DM
5	-	SD	-	-	-	8	Dead	DM

Abbreviations: CR, complete response; PR, partial response; SD, stable disease; PD, progressive disease; DM, distant metastasis; LR, locoregional relapse.

## DISCUSSION

PDTC is a rare type of thyroid malignancy that lacked standardized diagnostic criteria for a long time. Therefore, no large phase III trials have been performed on this entity. In the absence of randomized studies, the treatments for PDTC must be determined from retrospective series, which suggest a combination of surgical resection and adjuvant treatments (e.g., ^131^I therapy, EBRT and chemotherapy) [10, 16]. However, the effects of those adjuvant therapies remain under debate. A total of 91 patients (pT_1-4a_) with primary PDTC underwent surgical resection with or without adjuvant therapy at Memorial Sloan-Kettering Cancer Center from 1986 to 2009. With a median follow-up of 50 months, the 5-year overall survival and disease-specific survival were 62% and 66%, respectively. The 5-year locoregional and distant control rates were 81 and 59%, respectively [4]. That study demonstrated the advantage of surgery in PDTC. Ibrahimpasic et al. [15] also reported a retrospective study on 27 patients with PDTC presenting with gross extrathyroidal (pT_4a_) extension. All of the patients received aggressive surgery, and 77% of the patients had adjuvant therapy. Locoregional control was good, with 5-year local recurrence-free survival of 70% and 5-year overall survival of 47%.

However, PDTC shows a higher prevalence of laryngotracheal, esophageal or prevertebral space infiltration, or encasement of the carotid arteries or jugular veins, which cannot be resected even with aggressive surgery (i.e., total laryngectomy, tracheal resection and anastomosis, pharyngoesophagectomy with reconstruction, and vascular reconstruction). To date, no standardized treatment and follow-up strategy have been established for such patients. The use of EBRT in differentiated thyroid cancer was recommended by The Endocrine Surgery Committee of the American Head and Neck Society for patients with gross residual or unresectable locoregional disease, because it was reported to improve survival and locoregional control [17]. In a large retrospective study, 217 patients with differentiated thyroid carcinoma were classified as having gross residual disease. The 10-year locoregional recurrence-free survival was improved from 24% (treated without EBRT) to 63.4% (treated with EBRT) (*P* < 0.0001) [18]. Therefore, the lessons learned from patients with locally advanced, differentiated thyroid carcinoma can probably be applied to those with unresectable PDTC. Unfortunately, the available evidence does not show a strong association between EBRT and locoregional control or prolonged survival in PDTC [4, 6, 15, 19].

It was known that the total radiation dose was related to local control, but toxicities also increased with dose. Because of the special anatomical position, a compromise of target volume coverage was often considered in patients with thyroid carcinoma with the risk of spinal cord damage, which may somewhat account for the unsatisfactory results of EBRT in poorly differentiated thyroid carcinoma. Nutting et al. [20] performed a planning study on six consecutive patients with thyroid carcinoma to compare the effects of conventional radiotherapy, 3DCRT and IMRT. A dose of 60 Gy in 30 fractions was prescribed. IMRT plans achieved the goal dose to the PTV (*P* < 0.01 compared to 3DCRT (57.2 Gy)) and also reduced the spinal cord maximum dose to 40.7 Gy (*P* < 0.01). It seems that IMRT delivers a homogeneous higher dose to target volumes while lowering the dose to normal organs at risk. However, due to the small number of patients with poorly differentiated thyroid carcinoma who have received IMRT, no single institute has sufficient experience with the treatment effect.

Our preliminary results of IMRT for patients with T_4b_ tumors (unresectable disease) showed that the tolerance of patients was significantly improved, because four of the five patients received the goal dose of radiation (66 Gy). Only the patient who received 64 Gy discontinued the radiotherapy early because of esophagitis (Grade II), but the patient's dysphagia improved with medication to treat the symptoms. All five of the patients had stable disease after radiation. The use of IMRT seems to confer locoregional control. Specifically, 80% (4/5) of the patients had a partial response in 18 months, and 60% (3/5) of the patients had locoregional control (including 2 complete and 1 partial response) in 48 months. In total, 40% (2/5) of the patients had a complete response. Therefore, an 80% locoregional disease control rate (including complete remission, partial remission and stable disease) was achieved.

Despite the modest number of patients, it was confirmed that a stable overall survival and excellent locoregional control could be achieved through the IMRT-based therapy in unresectable PDTC. The main causes of disease-related death in PDTC were distant metastases and locoregional control. A previous multivariate analysis demonstrated the following poor prognostic factors: 1) age over 45 years, 2) tumor size over 4 cm, and 3) gross extrathyroidal extension [21, 22], which suggested that surgical resection is an independent predictor of survival, as reported by Ibrahimpasic et al. [4]. In this group of patients treated with IMRT, two of the patients presented at diagnosis with metastatic disease, and no other patients developed new metastases during the course of the disease. The direct causes of death were distant metastases (2/3 patients) and locoregional progression (1/3 patients).

The role of chemotherapy in PDTC is still unclear. One study examined the *in vitro* chemosensitivity of primary cultures of five PDTCs, and none showed a response to chemotherapy. Four of the tumors were resistant to all drugs examined: adriamycin, cisplatin, cyclophosphamide, etoposide and carboplatin [23]. In one recently published study, 13 patients with PDTC were treated with neoadjuvant chemotherapy between 1986 and 2005. Chemotherapy consisted of vinblastine, vinblastine with adriamycin or vinblastine with cisplatin, and a 38% response rate was reported [24]. Other reports demonstrated that an intensive chemotherapy regimen can aid in locoregional control by improving resectability or by reducing disease progression, suggesting its use in patients with unresectable tumors [25]. In recent years, new drugs, including paclitaxel and gemcitabine, have been used in patients with ATC, and long-term survival is expected to improve compared to those treated without paclitaxel or gemcitabine [26]. In our series, cisplatin was combined with paclitaxel or gemcitabine, but there was no clear correlation between the specific chemotherapy administered and outcome. Of course, there is a place for targeted therapy in PDTC, but at the present time, there has been little published on its use in poorly differentiated thyroid carcinoma. Therefore, further studies are necessary.

The main toxicities of the radiotherapy were dysphagia and esophagitis, which may have made the patients request to discontinue the radiotherapy early. In another study, thirteen patients with locally advanced thyroid cancer were recruited in a phase I study to demonstrate the safety of IMRT. In that study, no grade IV toxicity was observed, but 31% of patients experienced grade III dysphagia and 38.5% of patients experienced grade III dermatitis. Additionally, 31% patients required enteral feeding and 30% patients developed L’Hermitte's syndrome [27]. Compared to that study, the side effects in our study of IMRT were more mild or moderate, and the patients recovered rapidly with or without medication to manage their symptoms. None of the patients required tube feeding support or tracheostomy during radiotherapy. We must acknowledge that the dataset of our study is too small considering the number of variables that were evaluated, limiting the impact of our analyses.

In summary, IMRT-based therapy may be of benefit for locoregional control, with acceptable toxicity in patients with T_4b_ tumors (unresectable disease). Although the survival rates were worse than those that have been reported for resectable PDTC, the survival rate we observed is still acceptable (40%). The primary cause of death in our patients was distant metastases. Therefore, new therapies are still desperately needed.

## MATERIALS AND METHODS

### Patients and pretreatment evaluations

This study was approved by our institution's ethics committee (Fudan University Shanghai Cancer Center Institutional Review Board, reference number 090371-5). Between February 2011 and April 2012, we analyzed the outcomes of 5 unresectable PDTC patients treated with IMRT in our institution. There were 2 males and 3 females with a median age of 55 years (range: 43-76). All patients had histologically confirmed PDTC. Patient 4 and 5 had evidence of lung metastases before the start of treatment, and patient 3 presented with recurrent disease. The clinical characteristics of the five patients are listed in Table 3. The initial evaluation of the patients included medical history and physical examination, routine labs, computed tomography (CT) scans of the chest and neck, abdominal ultrasound, and bone scintigram. Additional investigations were performed only on those patients with suspicious findings. All patients in this study had advanced unresectable disease. The patient's disease was staged according to the seventh AJCC Cancer Staging Manual as follows: 3 were T4bN1bM0, and 2 were T4bN1bM1 (Table 3).

**Table 3:** Patients characteristics

Patient no.	Age/sex	Diagnosis	Stage	Size (cm)	Tracheal invasion	Esophageal invasion	Prevertebral space	Vessel encasement	Mediastinum	Nodal metastases	Distant metastases
1	64/F	Biopsy	T_4b_N_1b_M_0_	3.7*2.7	+	+	+	+	+	+	-
2	43/M	Biopsy	T_4b_N_1b_M_0_	6.5*6.5	+	+	+	+	+	+	-
3	49/F	Surgery /Biopsy	T_4b_N_1b_M_0_/Relapse	4.5*3.5	+	+	-	+	-	+	-
4	76/F	Biopsy	T_4b_N_1b_M_1_	6*6	+	+	+	+	+	+	lung
5	55/M	Biopsy	T_4b_N_1b_M_1_	9.6*7.9	+	+	+	-	-	+	lung

Abbreviations: M, male; F, female.

### Radiotherapy techniques

**Figure 2:**
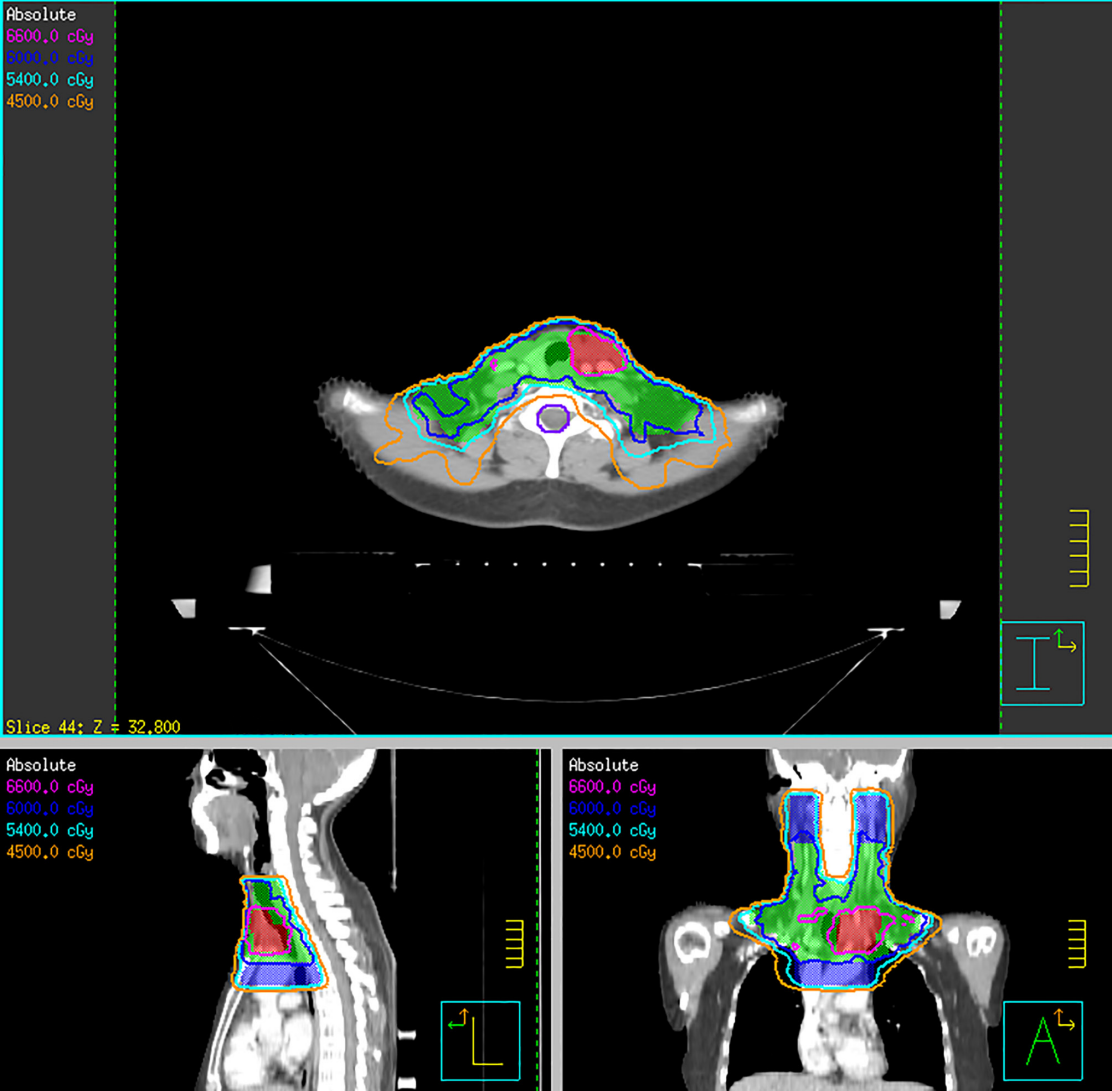
Isodose curve distributions for a representative patient with poorly differentiated thyroid carcinoma Shown are axial, coronal and sagittal slices representative of this patient's isodose curve distributions. Red contour represents planning target volume to 66 Gy, green contour represents planning target volume to 60 Gy, and blue contour represents planning target volume to 54 Gy. The spinal cord was spared with a sharp dose fall-off.

All 5 patients underwent a radiotherapy planning CT scan of the head and neck. The patients were immobilized using a thermoplastic mask in the supine position and had images taken at 5 mm intervals from the head to the level of the diaphragm. The treatment was performed with 6 MV photons. Gross tumor volume (GTV) included the primary tumor and metastatic lymph nodes that were discovered during clinical and imaging exams. Clinical target volume (CTV) included the thyroid area and regional lymph nodes, with elective treatment of bilateral level II through VI and mediastinal lymph nodes to the level of the carina. High-risk clinical target volume was defined as the thyroid bed plus any involved regional lymph nodes. A 5-mm margin with fine adjustment was added to convert GTV to planning target volume (PTV_g_). All patients were irradiated with 1 fraction daily, 5 days per week. The prescription doses were 66 Gy/33 fractions/6.6 weeks to the PTV_g_, 60 Gy to the high-risk clinical target volume plus 5-mm margin and 54 Gy to the low-risk clinical target volume. The organs at risk (OARs), such as the larynx and spinal cord, were constrained. The dose to the OARs was limited as followings: a <70 Gy maximum point dose and a <45 Gy mean dose for larynx, as well as a <45 Gy maximum point dose for spinal cord (Figure 2).

### Follow-up and assessment

After treatment, the patients were followed up every 3 months for the first 2 years after the completion of their treatment, then every 6 months for the following years. All patients were followed until death or until the time of analysis. The survival time was determined from the date of treatment to the time of death or the date of the most recent follow-up examination. The assessment of tumor response was based on CT imaging according to the Response Evaluation Criteria for Solid Tumors (RECIST) criteria [28]. IMRT-induced toxicities were scored according to the Acute and Late Radiation Morbidity Scoring Criteria of the Radiation Therapy Oncology Group (RTOG) [29], whereas systemic chemotherapy adverse effects were scored using the National Cancer Institute Common Toxicity Criteria (NCI CTCAE, version 3.0) [30].

## Abbreviations

ATC: Anaplastic thyroid carcinomas
CT: Computed tomography
CTV: Clinical target volume
EBRT: External beam radiotherapy
GTV: Gross tumor volume
IMRT: Intensity-modulated radiotherapy
OARs: Organs at risk
PDTC: Poorly differentiated thyroid carcinoma
PTV: Planning target volume
RTOG: Radiation Therapy Oncology Group
WDTC: Well-differentiated thyroid carcinomas
3DCRT: Three-dimensional conformal radiotherapy
